# Prevalence of New-Onset Atrial Fibrillation and Associated Outcomes in Patients with Sepsis: A Systematic Review and Meta-Analysis

**DOI:** 10.3390/jpm12040547

**Published:** 2022-03-30

**Authors:** Bernadette Corica, Giulio Francesco Romiti, Stefania Basili, Marco Proietti

**Affiliations:** 1 Department of Translational and Precision Medicine, Sapienza—University of Rome, 00161 Rome, Italy; bernadette.corica@uniroma1.it (B.C.); giuliofrancesco.romiti@uniroma1.it (G.F.R.); stefania.basili@uniroma1.it (S.B.); 2 Liverpool Centre for Cardiovascular Science, University of Liverpool and Liverpool Heart and Chest Hospital, Liverpool L10 0AD, UK; 3 Department of Clinical Sciences and Community Health, University of Milan, 20122 Milan, Italy; 4 Geriatric Unit, IRCCS Istituti Clinici Scientifici Maugeri, 20138 Milan, Italy

**Keywords:** atrial fibrillation, sepsis, septic shock, prognosis, meta-analysis

## Abstract

Background: New-onset atrial fibrillation (NOAF) is a common complication in patients with sepsis, although its prevalence and impact on outcomes are still unclear. We aim to provide a systematic review and meta-analysis on the prevalence of NOAF in patients with sepsis, and its impact on in-hospital mortality and intensive care unit (ICU) mortality. Methods: PubMed and EMBASE were systematically searched on 26 December 2021. Studies reporting on the prevalence of NOAF and/or its impact on in-hospital mortality or ICU mortality in patients with sepsis or septic shock were included. The pooled prevalence and 95% confidence intervals (CI) were calculated, as well as the risk ratios (RR), 95%CI and 95% prediction intervals (PI) for outcomes. Subgroup analyses and meta-regressions were performed to account for heterogeneity. Results: Among 4988 records retrieved from the literature search, 22 articles were included. Across 207,847 patients with sepsis, NOAF was found in 13.5% (95%CI: 8.9–20.1%), with high heterogeneity between studies; significant subgroup differences were observed, according to the geographical location, study design and sample size of the included studies. A multivariable meta-regression model showed that sample size and geographical location account for most of the heterogeneity. NOAF patients showed an increased risk of both in-hospital mortality (RR: 1.69, 95%CI: 1.47–1.96, 95%PI: 1.15–2.50) and ICU mortality (RR: 2.12, 95%CI: 1.86–2.43, 95%PI: 1.71–2.63), with moderate to no heterogeneity between the included studies. Conclusions: NOAF is a common complication during sepsis, being present in one out of seven individuals. Patients with NOAF are at a higher risk of adverse events during sepsis, and may need specific therapeutical interventions.

## 1. Introduction

As defined by the Third International Consensus Definitions for Sepsis and Septic Shock (Sepsis-3) [1], sepsis is a life-threatening organ dysfunction, caused by a dysregulated host response infection. Although mortality rates have decreased over the last decades, sepsis still imposes high morbidity and remains a major cause of death [2]. Cardiovascular complications, which often occur in these patients, are responsible for a significant proportion of the mortality [3]; among these, new-onset atrial fibrillation (NOAF) has been described as one of the most common complications. Inflammation, oxidative stress, electrolyte imbalance and iatrogenic factors (including the use of vasopressors) are deemed responsible for the increased risk of NOAF in patients with sepsis [4,5,6], although research on the pathophysiological link between these two diseases is still ongoing. However, it seems established that infections (particularly those affecting the lower respiratory tract) may act as a trigger for NOAF [7,8], and this observation was further confirmed during the COVID-19 pandemic [9].

Beyond the causes of its occurrence, NOAF imposes major challenges in the management of sepsis patients, which is still surrounded by uncertainties. Increased heart rate, worsening cardiac performance and hemodynamic instability, caused by a rapid ventricular response, often require additional treatment and longer hospitalizations [10,11]; furthermore, the role of anticoagulants for both short-term and long-term thromboembolic prevention is still debated, and represents an open question [6]. Finally, while an association between the onset of NOAF and increased mortality during sepsis has been described [12], the overall prevalence of NOAF in this clinical scenario is still unclear, as well as the magnitude of its impact on outcomes.

The aim of this systematic review and meta-analysis is to estimate the prevalence of NOAF in patients with sepsis, and to explore the association of NOAF with outcomes.

## 2. Materials and Methods

This systematic review was performed according to the Preferred Reporting Items for Systematic Reviews and Meta-Analyses (PRISMA) guidelines and recommendations (http://www.prisma-statement.org/, last accessed on 1 March 2022). The protocol was registered in the International Prospective Register of Systematic Reviews (PROSPERO), N. CRD42021227370.

### 2.1. Search Strategy

A systematic and comprehensive literature search was performed. PubMed and EMBASE databases were searched from inception to 26 December 2021. The search strategy was developed by two authors (G.F.R. and B.C.) and included a combination of key relevant terms related to the research question, including ‘sepsis’ and ‘atrial fibrillation’. The full search strategy can be found in the Supplementary Materials (Table S1).

### 2.2. Studies Selection

According to PRISMA guidelines, all records retrieved from the literature search were systematically and sequentially screened by two authors (G.F.R. and B.C.) independently, according to titles and abstracts. After the first screening phase, each included article was then assessed for full-text eligibility. Disagreements were resolved by collegial discussion.

### 2.3. Inclusion and Exclusion Criteria

Inclusion criteria were as follows: (i) studies reporting the prevalence of NOAF in patients with sepsis, and (ii) studies reporting outcomes (i.e., in-hospital mortality or ICU mortality) in sepsis patients, according to NOAF status. We also included studies reporting about patients with septic shock. Exclusion criteria were as follows: (i) studies with less than 50 sepsis patients included; (ii) conference abstracts, comments, editorials, case reports, systematic reviews, and meta-analyses; (iii) articles written in languages other than English. In the case of two or more studies based on the same cohort of patients, and exploring the same outcomes, we selected the study with the highest number of patients included, or the last published.

### 2.4. Data Extraction and Quality Assessment

Data from the included studies were extracted independently by two co-authors (G.F.R. and B.C.), through a standardized electronic form. Data on sample size, numbers of patients with NOAF and events of interest were extracted. When available, we also collected data about geographical location, study design, and relevant baseline characteristics (i.e., age, sex, percentage of patients treated in the ICU, hypertension, diabetes, congestive heart failure and history of stroke).

All included studies were independently evaluated by two co-authors (G.F.R. and B.C.) to assess the risk of bias. As we investigated two different outcomes (the prevalence of NOAF and outcomes according to NOAF status), we performed two separate evaluations of bias. For the first evaluation, we assessed all included studies that reported the prevalence of NOAF, using a customized version of the Newcastle–Ottawa Scale (NOS) for cross-sectional studies, composed of 5 items across three domains (selection, comparability and outcome), with a maximum of 5 points. Any study with a score ≤3 was categorized as having a high risk of bias. In the second evaluation, we assessed all studies reporting data on outcomes according to NOAF status, using an adapted version of the NOS for cohort studies [13]. The screening was composed of 8 items across three domains (selection, comparability and outcome). Any study with an NOS ≤6 was categorized as having a high risk of bias.

### 2.5. Outcomes Definition

Prevalence of NOAF was defined as the proportion of patients who presented with at least one episode of NOAF during sepsis. To improve our specificity, we selected and included only those studies that clearly focused on NOAF (e.g., through definition of “new-onset AF”, or with the exclusion of patients with a previous episode of AF).

We also investigated the impact of NOAF on the incidence of in-hospital mortality and ICU mortality. We decided to analyze these events separately, although their definition may overlap across studies; however, since their clinical meaning may differ, we chose to rely on the outcome definition given in the original studies.

### 2.6. Statistical Analysis

Prevalence of NOAF was pooled from each of the included studies using a generalized linear mixed model (a random intercept logistic regression model) [14].

The numbers of events and the total number of patients in each group of interest (NOAF versus patients without NOAF) were pooled and compared using a random-effects model. Pooled estimates were reported as risk ratios (RR), 95% confidence intervals (CI) and prediction intervals (PI). PI represents a predicted range of the true effect in an individual or a new study, and provides useful information on the variability of the effect in different clinical settings [15,16]. The inconsistency index (I^2^) was calculated to measure heterogeneity. According to pre-specified cut-offs, low heterogeneity was defined as an I^2^ <25%, moderate heterogeneity when I^2^ was between 25 and 75%, and high heterogeneity when I^2^ was >75%.

For each outcome, a sensitivity analysis was performed with a “leave-one-out” approach, in which all studies were iteratively removed one at a time, in order to analyze their influence on both pooled estimates and heterogeneity. As for prevalence of NOAF, we also performed several subgroup analyses, according to the geographical location of the study, study design, risk of bias and number of sepsis patients included in the original cohorts, and a cumulative analysis based on the year of publication. To further investigate potential sources of heterogeneity, we performed univariate and multivariable meta-regressions, using the aforementioned variables investigated in the subgroup analyses as covariates.

Publication bias for studies reporting outcomes according to NOAF was not assessed, since less than 10 studies were included for each outcome investigated.

All the statistical analyses were performed using R version 4.1.2 (R Foundation for Statistical Computing, Vienna, Austria, 2020), with the use of ‘meta’, ‘metafor’ and ‘dmetar’ [17] packages.

## 3. Results

A total of 4988 results were found from the literature search (609 from PubMed and 4379 from EMBASE). After the removal of duplicates, 4552 articles were screened and 118 were assessed for full-text eligibility. Finally, 22 articles were selected and included in the systematic review and meta-analysis [12,18,19,20,21,22,23,24,25,26,27,28,29,30,31,32,33,34,35,36,37,38]. The PRISMA flow-chart and reasons for studies’ exclusion are reported in Figure S1 in the Supplementary Materials.

### 3.1. Systematic Review of the Included Studies

Among the 22 studies selected, a total of 207,847 individuals with sepsis were included. The main characteristics of the included studies are reported in Table 1. Thirteen studies were held in North America [12,18,19,20,21,22,28,31,33,34,36,37,38], seven in Europe or the Middle East [23,25,26,27,29,32,35], and two in Asia [24,29]. Fourteen studies were retrospective [18,19,20,21,22,25,26,28,29,31,32,33,34,38], five were single-center observational [23,24,30,35,36], two studies were based on administrative databases [12,37], and one was a multicenter observational study [27]. As for the number of included patients with sepsis, 8 studies enrolled more than 1000 patients [12,18,19,22,25,31,36,37], 8 included between 100 and 1000 patients [20,21,26,27,28,29,33,38], and 6 enrolled less than 100 patients [23,24,30,32,34,35]. In 15 studies, only sepsis patients were included in the main analysis [12,18,19,21,23,25,26,27,28,29,33,34,35,37,38], while, in 7 studies, data were extracted for the subgroup of included sepsis patients [20,22,24,30,31,32,36].

Most of the studies were conducted in the ICU; one study was based on an internal medicine ward [32], and for two studies, based on administrative databases, the intensity of care was not reported [12,37].

As for the risk of bias, nine studies among those exploring prevalence were found to have a high risk of bias [18,20,22,24,26,32,35,36,38] (Table S2). Missing or incomplete reporting of baseline characteristics in patients with sepsis and low sample sizes were two of the most common concerns. Conversely, only one study [20] was considered to have a high risk of bias among those reporting outcomes according to NOAF presence (Table S3).

### 3.2. Prevalence of NOAF in Patients with Sepsis

**Figure 1:**
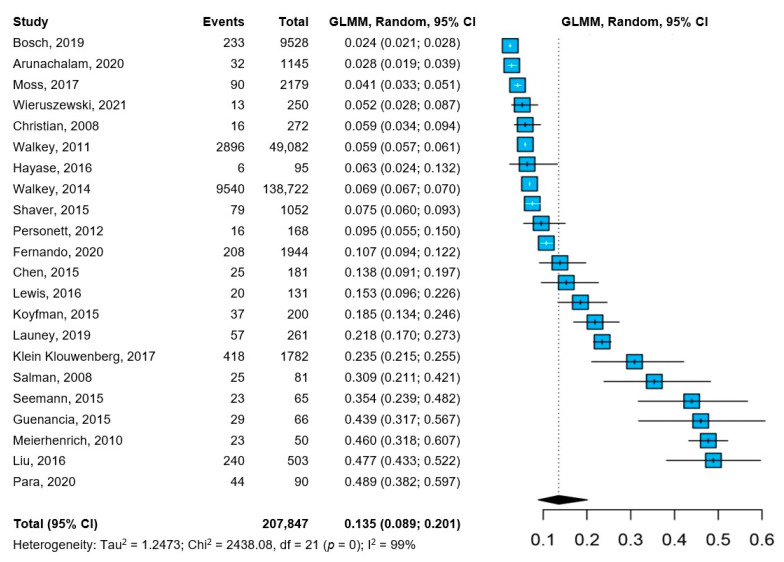
Pooled prevalence of NOAF in patients with sepsis [12,18,19,20,21,22,23,24,25,26,27,28,29,30,31,32,33,34,35,36,37,38]. Abbreviations: CI = confidence interval; GLMM = generalized linear mixed model; NOAF = new-onset atrial fibrillation; I^2^ = inconsistency index.

All the included studies reported about the prevalence of NOAF in patients with sepsis. The pooled prevalence of NOAF was 13.5% (95%CI: 8.9–20.1%), with high heterogeneity among the studies (Figure 1). The leave-one-out analysis, with the exclusion of one study at a time, showed little to no influence of individual studies, both in terms of pooled prevalence and between-study heterogeneity (Figure S2), and the cumulative analysis, based on the year of publication, showed substantially stable temporal estimates (Figure S3).

We performed several subgroup analyses, according to the baseline characteristics of the included studies (Table 2). Among the factors explored, significant subgroup differences were found according to geographical location (with lower prevalence reported in North American-based cohorts, compared to European/Middle East and Asian studies), study type (with higher prevalence of NOAF reported by observational studies, compared to retrospective and administrative-based studies) and the number of sepsis patients included in the original studies (with lower prevalence observed in studies with more than 1000 patients included, compared to those with 100 to 1000 patients, or less than 1000 patients with sepsis). No significant differences were observed according to the composition of the cohorts (only sepsis patients vs. sepsis defined as a subgroup of the whole cohort) or the risk of bias.

Univariate and multivariable meta-regression models are reported in Table 3. In the univariate meta-regressions, only geographical location and sample size significantly influenced the prevalence of NOAF in patients with sepsis; the final multivariable meta-regression model confirmed the independent impact of geographical location and sample size in influencing the prevalence of NOAF, accounting for most of the heterogeneity (R^2^ = 69.8%, *p* < 0.001).

### 3.3. In-Hospital and ICU Mortality

**Figure 2:**
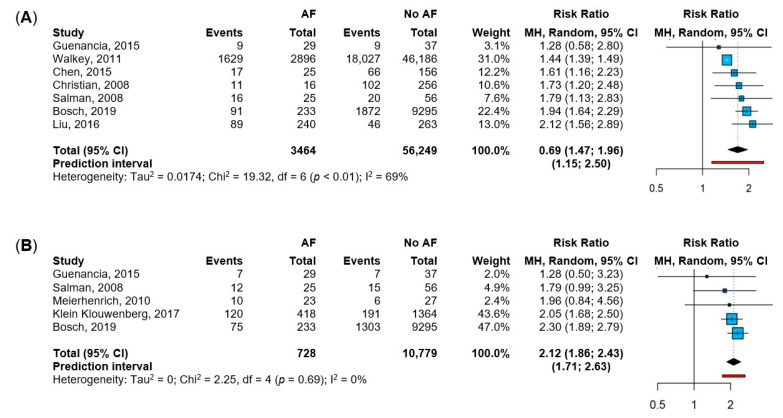
Impact of NOAF (new-onset atrial fibrillation) on in-hospital and ICU mortality. Legend: panel (**A**): in-hospital mortality [12,19,20,21,23,29,34]; panel (**B**): ICU (intensive care unit) mortality [19,23,25,30,34]. Abbreviations: AF = atrial fibrillation; MH = Mantel-Haenszel; CI = confidence interval; I2 = inconsistency index.

Seven studies [12,19,20,21,23,29,34] reported data about in-hospital mortality, while five studies [19,23,25,30,34] described ICU mortality (Figure 2). Patients with sepsis and NOAF showed a 1.7-fold higher risk of in-hospital mortality, with moderate heterogeneity between the studies, and a 95%PI between 15% and a 2.5-fold increased risk (Figure 2A). The sensitivity analysis according to the leave-one-out approach showed consistent results; however, the exclusion of the study by Walker et al. reduced the heterogeneity (I^2^ = 0%; Figure S4). As for ICU mortality, patients with NOAF were at a 2.1-fold higher risk compared to sepsis patients without NOAF, with no heterogeneity between the included studies, and a 95%PI between 1.7-fold and 2.6-fold increased risk (Figure 2B). Consistently, the leave-one-out analysis showed similar results when each study was excluded from the pooled estimate (Figure S5).

## 4. Discussion

In this systematic review and meta-analysis, including 207,849 adults with sepsis, we found that NOAF is a common complication, being present in one out of seven individuals. Patients with sepsis and NOAF were also exposed to a significantly higher risk of in-hospital and ICU mortality, underlining how the onset of AF may represent a key detrimental factor during the clinical course of sepsis.

Our meta-analysis—which is, to our knowledge, the first to report both pooled estimates for the prevalence of NOAF and the magnitude of its effect on outcomes—adds to the existing evidence on the tight relationship between AF and infections [39], these being among the most common conditions that may predispose patients to the onset of AF; moreover, previous reports have already identified infection-related AF as a risk factor for adverse outcomes [40], including during sepsis [41], and a recent meta-analysis has found consistent estimates of in-hospital mortality among patients with AF and sepsis, although without reporting estimates on the prevalence of NOAF in patients with sepsis [42].

### 4.1. Prevalence of NOAF Is Heterogeneous among Sepsis Patients

Patients presenting with AF during sepsis may be categorized into one of the following two main groups: those with previous episodes of AF (in which an acute infection may promote recurrence of the arrhythmia), and those without a previous history of overt AF (i.e., those with NOAF). These two groups may differ in terms of the incidence of sepsis-related AF, baseline characteristics, pathogenesis of the arrhythmia, and association between the AF episode and the incidence of adverse outcomes. In our study, we chose to only focus on NOAF, thus excluding cohorts in which patients with a previous history of AF were also included.

Notwithstanding, we found that the prevalence of NOAF was highly heterogeneous among the studies. Several factors may be responsible for the high between-study variance observed, including the severity of sepsis in the patients (with septic shock being associated with a higher incidence of NOAF) [43], study setting and design, definition of NOAF, and baseline characteristics of the patients included. Indeed, in our subgroup analyses, we found significant differences among different geographical locations, with European/Middle East cohorts reporting a five-fold higher prevalence, compared to North American-based studies. These differences may be attributable to differences in epidemiology and in the management of patients with sepsis across different countries; consistently, substantial geographical differences were already described for the incidence and mortality of sepsis [43]. Moreover, the study design may play a role, as we observed a higher prevalence of NOAF reported by observational studies and smaller cohorts. The definition of NOAF, as well as the active search for its presence in individuals with sepsis, may be an important factor to improve the diagnosis and management of these patients. In the final multivariable meta-regression model, the sample size and geographical location of the included studies were found to account for a significant proportion of the heterogeneity detected, consistent with these hypotheses.

### 4.2. NOAF Entails Worse Prognosis during Sepsis

The strong association between NOAF and mortality in patients with sepsis represents one key finding of our study. Patients with sepsis and NOAF showed an increased risk of both in-hospital and ICU mortality, compared to patients without NOAF; the PIs showed that this effect is likely to be confirmed in further studies, giving robustness to our results. Our study expands on the findings reported by a previous meta-analysis, which focused on in-hospital mortality [44], and confirms the results found in a broader cohort of critically ill patients [45].

Several mechanistic hypotheses may explain the association between NOAF and higher mortality in patients with sepsis. According to the supposed pathogenesis of arrythmia during infections [5], NOAF may represent an epiphenomenon of increased severity of the underlying sepsis, uncontrolled inflammation and cytokine storm, which may trigger coagulopathy, platelet activation and end-organ dysfunction [46,47]. Inflammation, particularly, also represents a postulated trigger of AF in other clinical settings, consistent with this hypothesis [48,49,50]. Unsurprisingly, inflammation was postulated as a potential therapeutic target in sepsis [51], and a rationale has also been described in patients with atrial fibrillation [52,53].

### 4.3. Future Perspectives on NOAF Management

The following major challenge remains in the management of AF in patients with sepsis: beyond the risk of mortality, an increased rate of stroke has also been described in these patients [12], with an unclear risk–benefit profile of anticoagulation therapy [54,55]. A retrospective cohort study, based on administrative data, was unable to find a significant reduction in thromboembolic risk in sepsis patients with AF, who were treated with parenteral anticoagulation, while a significant increase in the risk of clinically significant bleeding was found [54]. However, further studies are clearly required to shed light on the role of anticoagulation in this clinical scenario. Similarly, the optimal therapy for heart rate control is still debated [10,56]. Consistently, no specific recommendations were made for patients with sepsis and AF in the recent 2020 guidelines for the management of AF, issued by the European Society of Cardiology [57]. Further studies are urgently needed to clarify the best management strategies for the prevention of adverse events in patients with sepsis and NOAF; until this evidence is available, awareness of the increased risk of mortality and tailored approaches are strongly recommended.

### 4.4. Limitations

Our study has several limitations. First, a significant number of studies reported the prevalence and outcomes of NOAF patients in a general critically ill population, and data on the sepsis subgroup were extracted; this limited our ability to gather data on the baseline characteristics of the sepsis patients, which may have influenced the results of our analysis. However, several subgroup analyses showed significant differences between key groups of patients with sepsis, and low or moderate heterogeneity was detected across studies reporting outcomes in patients with NOAF. Relatedly, we were not able to assess the role of treatments (including anticoagulants) on the risk of outcomes, due to data availability, and further specifically designed studies are required to evaluate these open questions. Since data on the severity of sepsis (i.e., the proportion of patients with septic shock) were unavailable in a consistent proportion of the studies included, we did not perform a subgroup analysis; accordingly, it is possible that the prevalence of NOAF may be influenced by the underlying severity of sepsis. Furthermore, although most studies were conducted in an ICU setting, it is possible that some part of the heterogeneity may be due to the inclusion of cohorts from different clinical settings. Moreover, the inclusion of observational and retrospective studies may have led to potential bias in the definition of NOAF and its impact on outcomes; however, all the leave-one-out sensitivity analyses gave consistent results, compared to the principal model.

## 5. Conclusions

NOAF is a common complication in patients with sepsis, being present in almost one out of seven subjects, and is associated with an increased risk of in-hospital death and ICU mortality. Further studies are warranted to clarify the best management strategy for patients with sepsis and NOAF.

## Figures and Tables

**Table 1:** Characteristics of the studies included in the systematic review.

Study	Region	Design	Setting	Sepsis (*n*)	NOAF (*n*)	Age (Y)	Females (%)	Septic Shock (%)	HTN (%)	DM (%)	Outcomes Included ǂ
Arunachalam 2020 [18]	North America	Retrospective	ICU	1145	32	NR	NR	NR	NR	NR	None
Bosch 2019 [19]	North America	Retrospective	ICU	9528	233	66.5 (16.5)	46.4	NR	NR	NR	In-hospital mortality, ICU mortality
Chen 2015 [20]	North America	Retrospective	ICU	181	25	NR	NR	100	NR	NR	In-hospital mortality
Christian 2008 [21]	North America	Retrospective	ICU	272	16	62.4 (16.3)	NR	NR	NR	NR	In-hospital mortality
Fernando 2020 [22]	North America	Retrospective	ICU	1944	208	NR	NR	66.0	NR	NR	None
Guenancia 2015 [23]	Europe	Observational single center	ICU	66	29	65.1 (14.4)	36.4	100	51.5	21.2	In-hospital mortality
Hayase 2016 [24]	Asia	Observational single center	ICU	95	6	63.5 (15.1)	32.6	NR	NR	NR	None
Klein Klouwenberg 2017 [25]	Europe	Retrospective	ICU	1782	418	66 *	41.9	29.9	NR	16.7	ICU mortality
Koyfman 2015 [26]	Middle East	Retrospective	ICU	200	37	NR	NR	NR	NR	NR	None
Launey 2019 [27]	Europe	Observational multicenter	ICU	261	57	63.9 (14.1)	40.6	100	59.2	15.3	None
Lewis 2016 [28]	North America	Retrospective	ICU	131	20	61.6 (13.7)	51.9	NR	71.7	40.4	None
Liu 2016 [29]	Asia	Retrospective	ICU	503	240	73.2 (14.0)	38.4	NR	59.6	36.4	In-hospital mortality
Meierheinrich 2010 [30]	Europe	Observational single center	ICU	50	23	66 *	34	100	56	NR	ICU mortality
Moss 2017 [31]	North America	Retrospective	ICU	2179	90	NR	NR	NR	NR	NR	None
Para 2020 [32]	Europe	Retrospective	Non-ICU	90	46	NR	NR	NR	NR	NR	None
Personett 2012 [33]	North America	Retrospective	ICU	168	16	44.4 (33.7)	44.6	100	NR	25.6	None
Salman 2008 [34]	North America	Retrospective	ICU	81	25	59.2 (14.5)	43.0	66.7	40.0	23.0	In-hospital mortality, ICU mortality
Seemann 2015 [35]	Europe	Observational single center	ICU	65	23	NR	44.6	100	35.4	20.0	None
Shaver 2015 [36]	North America	Observational single center	ICU	1052	79	NR	NR	NR	NR	NR	None
Walkey 2014 [37]	North America	Administrative database	NR	138,722	9540	80 (7.6)	57.5	NR	90.5	55.1	None
Walkey 2011 [12]	North America	Administrative database	NR	49,082	2896	68.5 (16.4)	48.3	NR	49.8	33.6	In-hospital mortality
Wieruszewski 2021 [38]	North America	Retrospective	ICU	250	13	NR	46.8	100	NR	NR	None

Legend: Data are presented as mean (SD) and percentage if not specified; ǂ among those investigated in the meta-analysis (in-hospital mortality and ICU mortality); * median value for the subgroup of patients with NOAF. Abbreviations: NOAF = new-onset atrial fibrillation; Y = years; HTN = hypertension; DM = diabetes mellitus; ICU = intensive care unit; NR = not reported.

**Table 2:** Subgroup analysis of NOAF prevalence in patients with sepsis.

Subgroups	Number of Studies	Pooled Prevalence	95% CI	I^2^
Geographical Location (*p* for subgroup differences ≤ 0.001)				
North America	12	7.3	5.0–10.6	97.6
Europe/Middle East	7	32.1	23.8–41.6	89.9
Asia	2	20.3	3.9–61.9	97.3
Study Type (*p* for subgroup differences ≤ 0.001)				
Administrative database	2	6.4	5.7–7.1	98.2
Observational	6	22.2	11.2–39.2	96.5
Retrospective	14	12.1	7.0–20.1	99.2
Sample size (*p* for subgroup differences ≤ 0.001)				
More than 1000 patients	8	6.3	3.8–10.3	99.4
100–1000 patients	8	14.4	8.5–23.5	97.2
Less than 100 patients	6	32.4	19.5–48.8	86.1
Composition of the cohort (*p* for subgroup differences = 0.889)				
Only sepsis patients	15	13.3	7.9–21.4	99.3
Sepsis as a subgroup	7	14.1	6.6–27.6	97.6
Risk of Bias (*p* for subgroup differences = 0.649)				
Low Risk	13	14.6	8.3–24.5	99.4
High Risk	9	12.1	6.4–21.6	96.5

Abbreviations: CI = confidence interval; I^2^ = inconsistency index.

**Table 3:** Univariate and multivariable meta-regression analysis of NOAF prevalence.

Variable	Coefficient	Standard Error	Lower 95%CI	Upper 95%CI	*p*	R^2^
Univariate Analysis						
Study Type					0.211	0.139
Administrative (ref.)	-	-	-	-		
Retrospective	0.706	0.785	−0.938	2.349		
Observational	1.432	0.853	−0.353	3.217		
Geographical Location					<0.001	0.576
North America (ref.)	-	-	-	-		
Europe/Middle East	1.804	0.354	1.064	2.545		
Asia	1.282	0.586	0.055	2.509		
Sample Size					0.001	0.489
More than 1000 (ref.)	-	-	-	-		
100–1000	0.914	0.408	0.060	1.768		
Less than 100	1.962	0.447	1.027	2.897		
Composition of the cohort					0.890	0.000
Only sepsis patients (ref.)	-	-	-	-		
Sepsis as a subgroup	0.073	0.521	−1.013	1.159		
Risk of Bias					0.661	0.001
High Risk (ref.)	-	-	-	-		
Low Risk	0.219	0.491	−0.807	1.243		
Multivariable Analysis					<0.001	0.698
Sample Size						
More than 1000 (ref.)	-	-	-	-	-	
100–1000	0.646	0.334	−0.058	1.350	0.070	
Less than 100	1.136	0.424	0.242	2.030	0.016	
Geographical Location						
North America (ref.)	-	-	-	-	-	
Europe/Middle East	1.304	0.358	0.549	2.059	0.002	
Asia	0.788	0.539	−0.350	1.926	0.162	

Abbreviations: CI = confidence interval; ref. = reference.

## Data Availability

The data that support the findings of this study are available from the corresponding author, upon reasonable request, and after approval of all other co-authors.

## Supplementary Materials

The following supporting information can be downloaded at: www.mdpi.com/article/10.3390/jpm12040547/s1. Table S1: Full Search Strategy; Table S2: Bias Assessment—NOS for incidence of new onset Atrial Fibrillation; Table S3: Bias Assessment—NOS for outcomes according to new onset Atrial Fibrillation; Figure S1: PRISMA Flow-Chart of the Study; Figure S2: Leave one out analysis for NOAF Prevalence; Figure S3: Cumulative Meta-Analysis of Prevalence of NOAF based on Study Publication Year.; Figure S4: Leave one out analysis for in-hospital mortality according to NOAF; Figure S5: Leave one out analysis for ICU mortality in patients with NOAF. References [12,18,19,20,21,22,23,24,25,26,27,28,29,30,31,32,33,34,35,36,37,38] are cited in the Supplementary Materials.
