# Supplementary material for: Prevalence of New-Onset Atrial Fibrillation and Associated Outcomes in Patients with Sepsis: A Systematic Review and Meta-Analysis
Source: J Pers Med. 2022 Mar 30;12(4):547. doi: 10.3390/jpm12040547 (PMC9026551; doi:10.3390/jpm12040547)
Supplement: Supplementary file 1 [file jpm-12-00547-s001.zip › jpm-1642909 - Supplem. Mat - Conversion.pdf]

Journal of Personalized Medicine

MDPI

Supplementary Materials

Table S1. Full Search Strategy.

PubMed
#1 sepsis"[MeSH Terms] OR "sepsis"[All Fields] OR "septicaemias"[All Fields] OR "septicemias"[All Fields] OR "septicaemia"[All Fields] OR "septicemia"[All Fields]
#2 "bacteraemia"[All Fields] OR "bacteremia"[MeSH Terms] OR "bacteremia"[All Fields] OR "bacteraemias"[All Fields] OR "bacteremias"[All Fields]
#3 "septic"[All Fields] OR "septics"[All Fields]
#4 #1 OR #2 OR #3
#5 ("atrial fibrillation"[MeSH Terms] OR ("atrial"[All Fields] AND "fibrillation"[All Fields])) OR "atrial fibrillation"[All Fields]
#6 #4 AND #5
**EMBASE**
#1 'sepsis'/exp OR 'sepsis' OR 'septicemia' OR 'bacteremia' OR septic
#2 'atrial fibrillation'
#3 #1 AND #2

Table S2. Bias Assessment—NOS for incidence of new onset Atrial Fibrillation.

Study	Selection (2)	Comparability (1)	Outcome (2)	Total (5)
Arunachalam 2020* [18]	1	0	1	2
Bosch 2019 [19]	2	1	2	5
Chen 2015* [20]	1	0	1	2
Christian 2008* [21]	2	1	1	4
Fernando 2020* [22]	1	0	1	2
Guenancia 2015** [23]	1	1	2	4
Hayase 2016*,** [24]	1	0	1	2
Klein Klouwenberg 2017 [25]	2	1	2	5
Koyfman 2015* [26]	2	0	1	3
Launey 2019*** [27]	1	1	2	5
Lewis 2016 [28]	2	1	2	5
Liu 2016 [29]	2	1	2	5
Meierhenrich 2010 [30]	1	1	2	4
Moss 2017* [31]	2	1	1	4
Para 2020*,** [32]	0	0	1	1
Personett 2012*,# [33]	2	1	1	4
Salman 2008** [34]	1	1	2	4
Seemann 2015**,# [35]	1	1	1	3
Shaver 2015* [36]	1	1	1	3
Walkey 2014 [37]	2	1	2	5
Walkey 2011# [12]	2	1	1	4
Wieruszewski 2021*,# [38]	1	1	1	3

Legend: *No/missing baseline charateristics/information for the whole cohort of sepsis patients; **Sample size less than 100 patients. ***Selected african/american patients. #Possible bias in definition of noAF NOS = Newcastle-Ottawa Scale.

Table S3. Bias Assessment—NOS for outcomes according to new onset Atrial Fibrillation.

Study	Selection (4)	Comparability (2)	Outcome (3)	Total (9)
Bosch 2019 [19]	4	2	3	9
Chen 2015* [20]	3	0	3	6
Christian 2008* [21]	3	1	3	7
Guenancia 2015 [23]	4	2	3	9
Klein Klouwenberg 2017 [25]	4	2	3	9
Liu 2016 [29]	4	2	3	9
Meierhenrich 2010 [30]	4	1	3	8
Salman 2008 [34]	4	1	3	8
Walkey 2011# [12]	3	2	3	8

Legend: *No/Missing baseline charateristics/informations for sepsis patients; #Possible bias in definition of noAF NOS= Newcastle-Ottawa Scale.

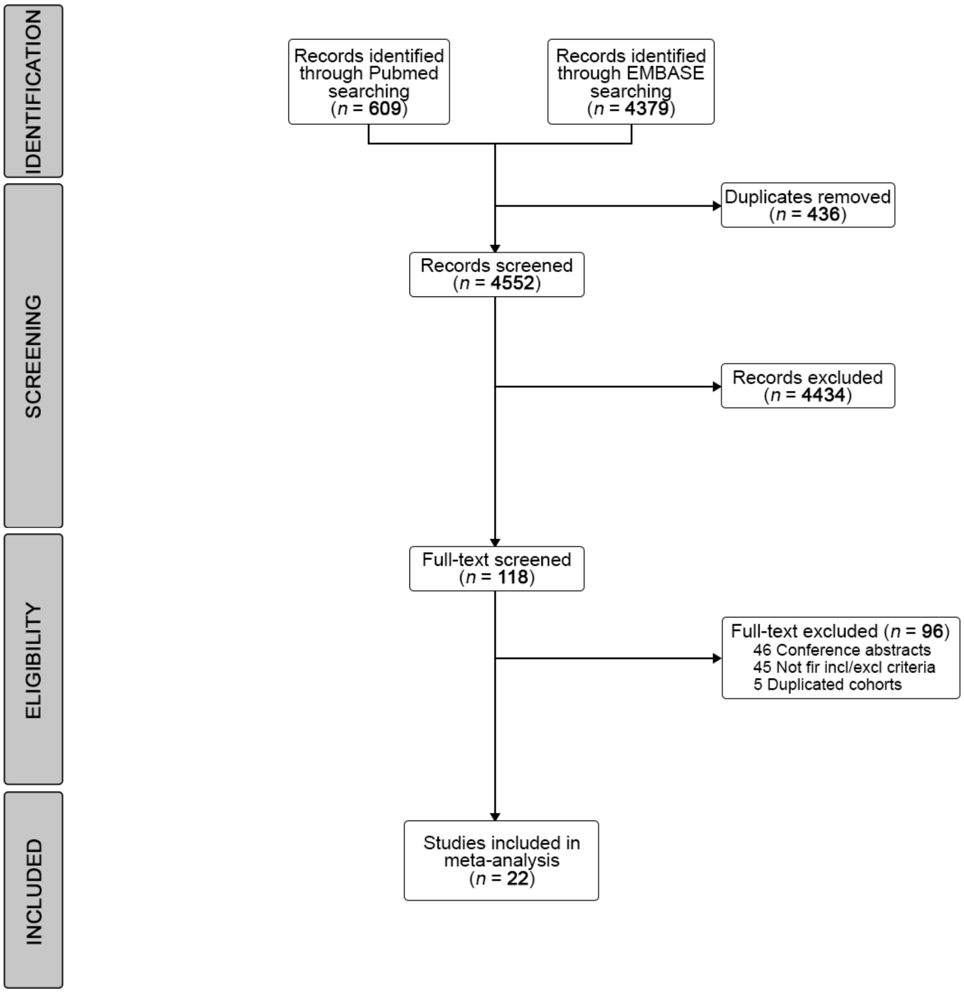

Figure S1. PRISMA Flow-Chart of the Study.

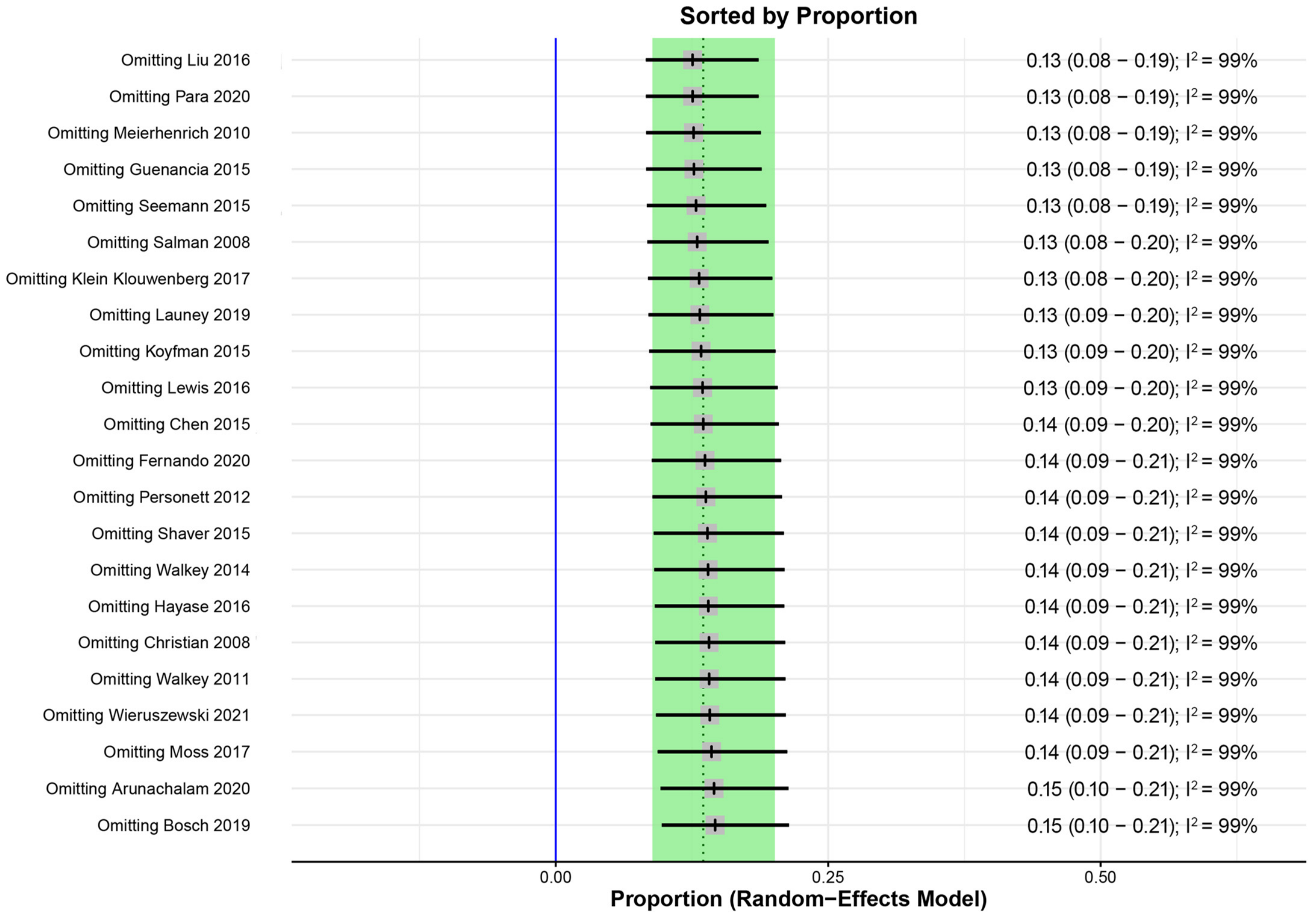

Figure S2. Leave one out analysis for NOAF Prevalence [12,18–38]. Abbreviation: I^2 = inconsistency index.

Study	GLMM, Random, 95% CI
Adding Christian 2008 (k = 1)	0.059 (0.036; 0.094)
Adding Salman 2008 (k = 2)	0.141 (0.040; 0.394)
Adding Meierhenrich 2010 (k = 3)	0.220 (0.074; 0.501)
Adding Walkey 2011 (k = 4)	0.160 (0.057; 0.375)
Adding Personett 2012 (k = 5)	0.144 (0.062; 0.302)
Adding Walkey 2014 (k = 6)	0.127 (0.061; 0.248)
Adding Chen 2015 (k = 7)	0.128 (0.068; 0.228)
Adding Guenancia 2015 (k = 8)	0.153 (0.081; 0.272)
Adding Koyfman 2015 (k = 9)	0.156 (0.089; 0.261)
Adding Seemann 2015 (k = 10)	0.171 (0.100; 0.275)
Adding Shaver 2015 (k = 11)	0.159 (0.096; 0.252)
Adding Hayase 2016 (k = 12)	0.148 (0.091; 0.232)
Adding Lewis 2016 (k = 13)	0.148 (0.094; 0.225)
Adding Liu 2016 (k = 14)	0.165 (0.104; 0.251)
Adding Klein Klouwenberg 2017 (k = 15)	0.169 (0.110; 0.250)
Adding Moss 2017 (k = 16)	0.155 (0.100; 0.233)
Adding Bosch 2019 (k = 17)	0.140 (0.088; 0.216)
Adding Launey 2019 (k = 18)	0.144 (0.093; 0.216)
Adding Arunachalam 2020 (k = 19)	0.132 (0.084; 0.202)
Adding Fernando 2020 (k = 20)	0.131 (0.085; 0.196)
Adding Para 2020 (k = 21)	0.141 (0.092; 0.211)
Adding Wieruszewski 2021 (k = 22)	0.135 (0.089; 0.201)
**Total (95% CI)**	**0.135 (0.089; 0.201)**

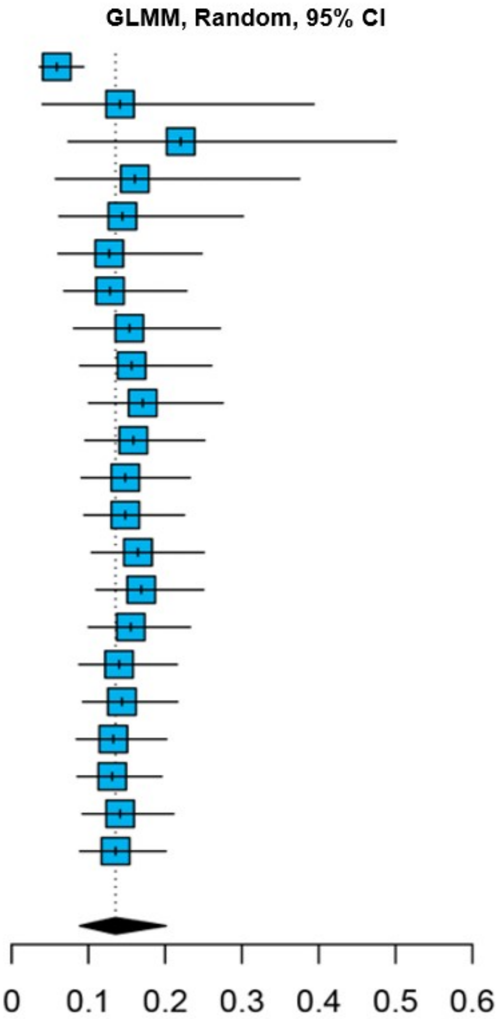

Figure S3. Cumulative Meta-Analysis of Prevalence of NOAF based on Study Publication Year [12,18–38]. Abbreviations: GLMM = generalized linear mixed model; CI = confidence interval.

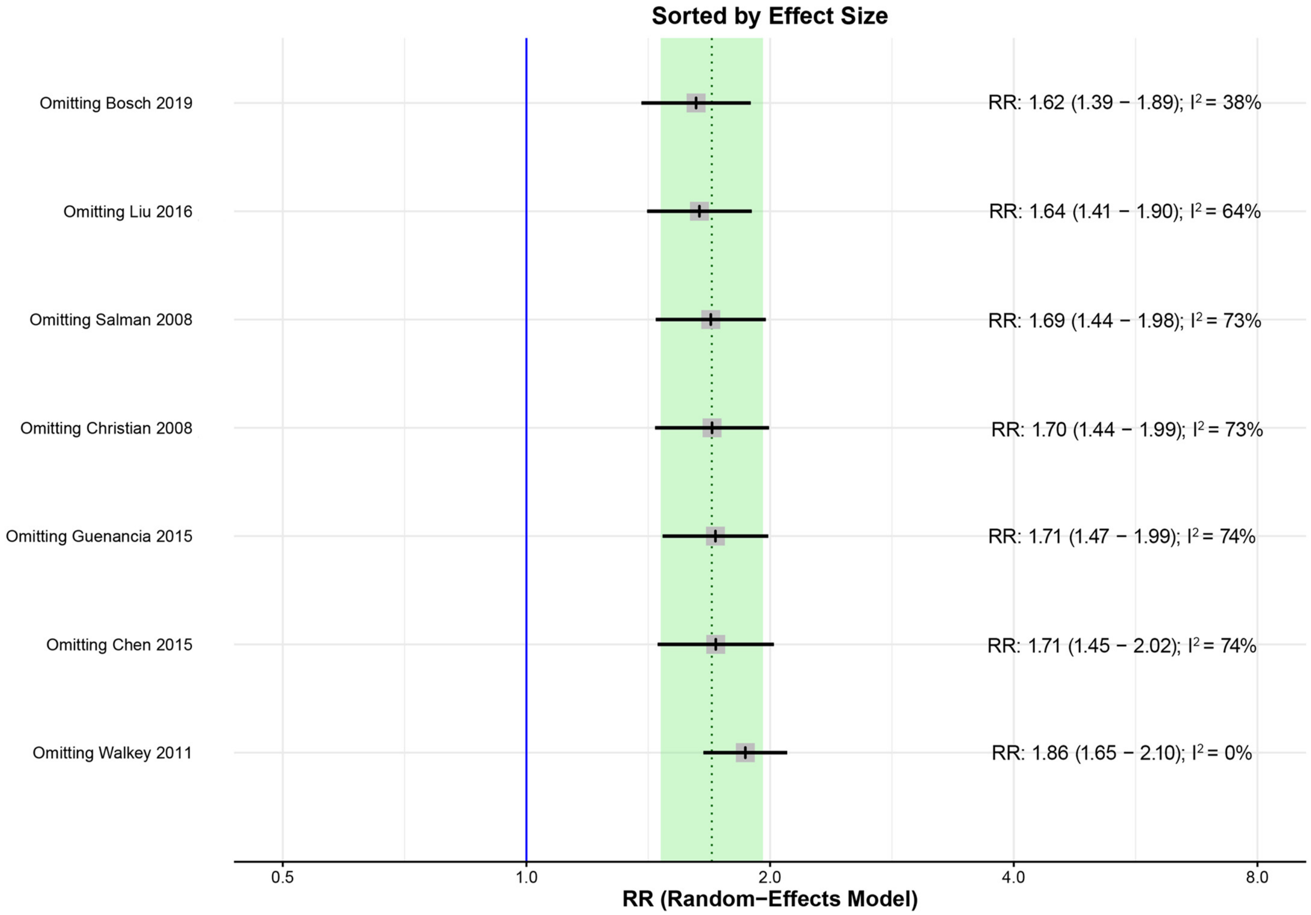

Figure S4. Leave one out analysis for in-hospital mortality according to NOAF [12,19–21,23,29,34]. Abbreviation: I^2 = inconsistency index, RR = Risk Ratio.

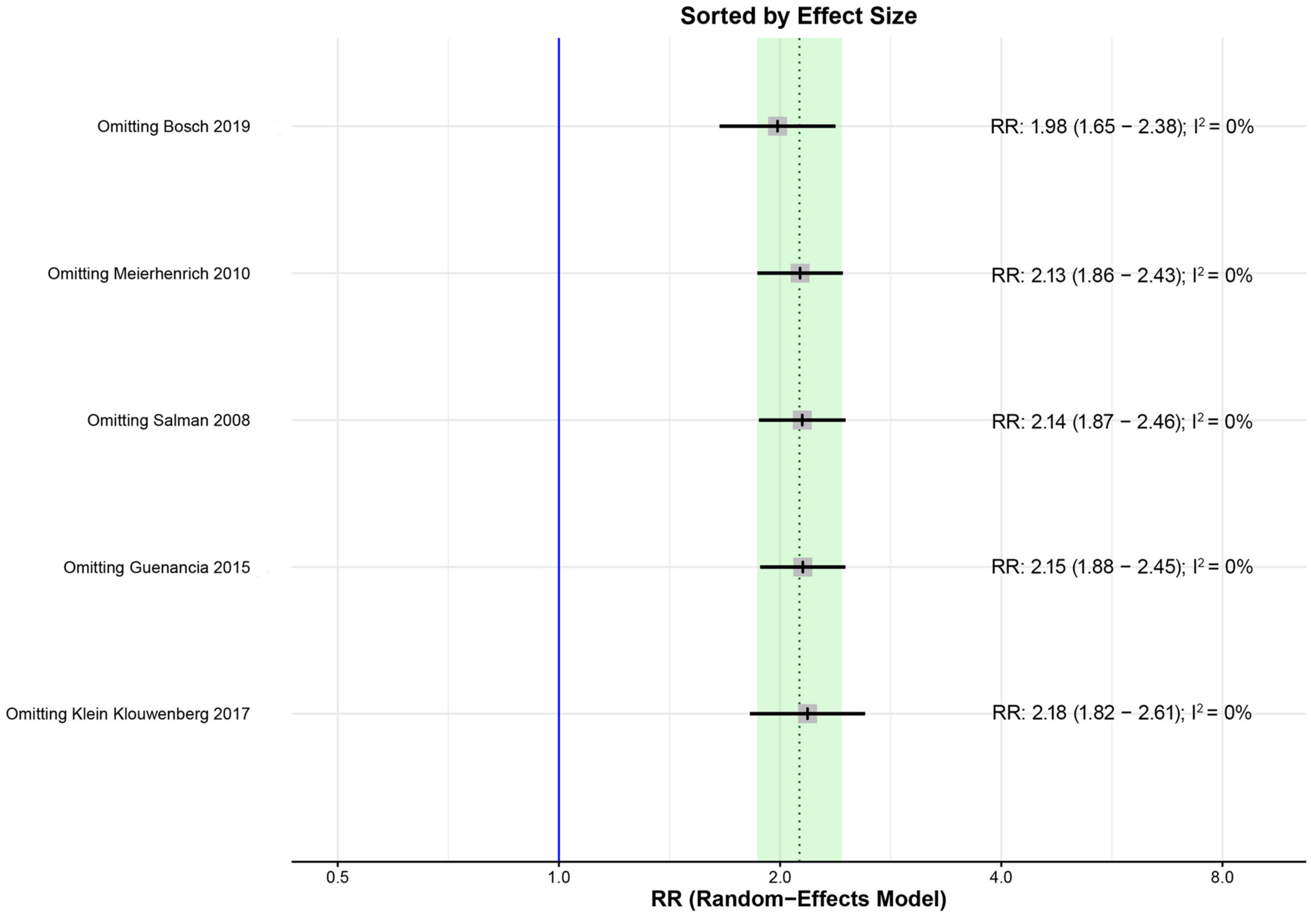

Figure S5. Leave one out analysis for ICU mortality in patients with NOAF [19,23,25,30,34]. Abbreviation: I^2 = inconsistency index, RR = Risk Ratio.
